# Secondary ocular hypertension as an adverse effect of treatment with intravitreal dexamethasone implant: A retrospective Swedish cohort study

**DOI:** 10.1111/aos.17475

**Published:** 2025-03-05

**Authors:** Imadeddin Abu Ishkheidem, Martin Breimer, Saba Kamal, Madeleine Zetterberg, Abbas Al‐Hawasi, Marita Andersson Grönlund

**Affiliations:** ^1^ Department of Clinical Neuroscience, Institute of Neuroscience and Physiology Gothenburg University Gothenburg Sweden; ^2^ Department of Ophthalmology Region Västra Götaland, Sahlgrenska University Hospital Gothenburg Sweden; ^3^ Department of Pharmacy Uppsala University Uppsala Sweden; ^4^ The Swedish Digital Ophthalmology Centre Linkoping Sweden; ^5^ Department of Ophthalmology, Faculty of Medicine and Health Örebro University Orebro Sweden

**Keywords:** diabetic macular oedema, intraocular pressure, intravitreal dexamethasone implant, ocular hypertension, retinal vein occlusion

## Abstract

**Purpose:**

To evaluate the incidence and risk factors for secondary ocular hypertension (SOHT) following intravitreal dexamethasone implants (Ozurdex®) in patients with diabetic macular oedema (DME) and macular oedema secondary to retinal vein occlusion (RVO) in a Swedish cohort.

**Methods:**

This retrospective study included 309 eyes from 249 patients treated with Ozurdex® at Sahlgrenska University Hospital, Mölndal, Sweden, from 1 January 2016 to 31 December 2023. Electronic medical records were reviewed for data including patient demographics, number of injections, incidence of and treatment modalities for SOHT and rate of Ozurdex® discontinuation.

**Results:**

Of the 309 eyes, 217 (70.2%) were in the DME group and 92 (29.8%) in the RVO group. Overall, 117 eyes (37.9%) developed SOHT (intraocular pressure (IOP) ≥25 mmHg or a rise of ≥10 mmHg from baseline); this included 77 of 217 DME eyes (35.5%) and 40 of 92 RVO eyes (43.5%). Men were more than twice as likely as women to develop SOHT (adjusted odds ratio [aOR]: 2.53, *p* < 0.001). Each unit increase in baseline IOP was associated with an 8% increase in the odds of SOHT (aOR: 1.08 *p* = 0.025). Of all eyes, 30.5% received IOP‐lowering treatment, primarily prostaglandins. None required invasive surgery.

**Conclusion:**

Our finding that 30.5% of eyes received IOP‐lowering treatment confirms that SOHT is a prevalent complication following intravitreal dexamethasone implants. Male gender and higher baseline IOP were key indicators for developing SOHT after Ozurdex® treatment, emphasizing the need for vigilant monitoring. Most cases were managed with IOP‐lowering eye drops, indicating that while common, SOHT is typically manageable without invasive interventions.

## 1 INTRODUCTION

Ozurdex® is an intravitreal dexamethasone implant that is widely employed in the management of various retinal diseases, including diabetic macular oedema (DME) and macular oedema secondary to retinal vein occlusion (RVO) (Boyer et al., 2014; Soliman et al., 2023). These implants, which release dexamethasone for up to 6 months, offer the advantage of sustained drug release, potentially improving therapeutic outcomes by maintaining optimal drug concentrations over extended periods (Chang‐Lin et al., 2011). Ozurdex® 0.7 mg was approved by the Food and Drug Administration (FDA) in June 2009 and obtained European marketing authorization in July 2010 for the treatment of DME and macular oedema due to RVO. However, while the efficacy of dexamethasone in reducing retinal inflammation and macular oedema is well documented, the treatment can have adverse effects, the most significant being secondary ocular hypertension (SOHT) (Malclès et al., 2017).

Secondary ocular hypertension following intravitreal steroid administration is a well‐recognized complication that may precipitate the onset of glaucoma, a condition that means progressive irreversible visual field loss and optic nerve damage if not properly managed (Gordon et al., 2002). Studies suggest variable incidence rates of SOHT post‐dexamethasone implantation (Fraser‐Bell et al., 2023; Malclès et al., 2017; Rajesh et al., 2020; Sweta et al., 2022), underscoring the need for a thorough understanding of its risk factors and long‐term consequences. Despite this, the pathophysiology underlying steroid‐induced SOHT is not fully elucidated, and the factors contributing to susceptibility among individuals remain incompletely defined (Roberti et al., 2020).

This retrospective cohort study aimed to enhance our understanding of the incidence of SOHT in patients treated with dexamethasone implants at Sahlgrenska University Hospital, Mölndal, Sweden, reflecting 8 years of clinical practice in the second‐largest ophthalmology unit in Sweden. Patient demographics, treatment modalities and outcomes were investigated in order to identify potential risk factors associated with the development of SOHT. The results of this research are intended to guide clinicians in optimizing safety and efficacy when employing intravitreal dexamethasone implants in the treatment of retinal diseases.

## 2 METHODS

### 2.1 Study design and setting

This retrospective cohort study was conducted at Sahlgrenska University Hospital, Mölndal, Sweden, between 1 January 2016 and 31 December 2023. Notably, 2016 marked the introduction of Ozurdex® in our clinic, allowing for the inclusion of all eligible patients treated with Ozurdex® since its inception. The research protocol adhered to the ethical standards of the Declaration of Helsinki and received approval from the Swedish Ethical Review Authority (ref: 2023‐06750‐01).

### 2.2 Participants

The study included 249 patients (309 eyes) who met the following criteria.

- **Inclusion criteria:**
  1. Patients diagnosed with DME or macular oedema secondary to retinal vein occlusion (RVO).
  2. Patients who received one or more intravitreal dexamethasone implants (Ozurdex®) during the study period, spanning from January 2016 to December 2023.
- **Exclusion criteria:**
  1. Cases where Ozurdex® implants were administered for indications other than DME or macular oedema secondary to RVO (e.g. uveitis, postoperative cystoid macular oedema) were excluded due to the limited number of such patients, which would preclude statistically meaningful conclusions for these specific conditions.
  2. Patients with incomplete medical records ensure the accuracy and integrity of data analysis.

### 2.3 Data collection

Data were meticulously extracted from electronic patient medical records at Sahlgrenska University Hospital and the Swedish Macular Registry. This comprehensive approach ensured that all relevant patient data were captured and verified across multiple systems, thereby enhancing data integrity and minimizing the risk of missing information. Variables collected included patient demographics, the number of dexamethasone injections received, the incidence of and treatment modalities for SOHT and reasons for treatment discontinuation. SOHT was precisely defined as an intraocular pressure (IOP) ≥25 mmHg or a rise of ≥10 mmHg from the baseline measurement, in alignment with the criteria used in the SAFODEX study (Malclès et al., 2017). IOP measurements were predominantly obtained using rebound tonometry (iCare IC100, Icare Finland Oy) as this is the standard routine in our clinic, reflecting real‐world clinical practice. In cases where the measured IOP was borderline or required confirmation, some clinicians opted to double‐check using Goldmann applanation tonometry. However, due to the high patient volume, iCare tonometry remained the primary method for IOP measurement during the study period.

The routine clinical algorithm for IOP monitoring, as part of the Ozurdex® treatment protocol, involved measurement at 4 weeks and 8 weeks after each Ozurdex® injection. Subsequent measurements were performed as deemed necessary by the treating clinician, based on the patient's clinical status and response to treatment. It is important to note that in real‐world settings, visits do not always occur exactly as planned; however, this protocol represented the routine followed in the clinic during the study period.

### 2.4 Data analysis

Counts and percentages were used for categorical variables, and measures of central tendency and variability (mean, standard deviation, median, min, max) were applied to continuous variables.

The core of the analysis utilized generalized estimating equations (GEE) with a logit function to explore the impacts of baseline IOP and the total number of Ozurdex® injections on the likelihood of SOHT, treatment provided for Ozurdex‐induced SOHT and discontinuation of treatment with Ozurdex®. The variables tested as independent predictors for the studied outcomes were age, age category (≤60 years vs. >60 years), sex, baseline IOP, baseline IOP category (<15 mmHg vs. ≥15 mmHg), total number of Ozurdex® injections and the number of injections leading to SOHT or total number if not leading to SOHT.

Most of the models employed a compound symmetry correlation structure, with a few exceptions where parameter estimation issues required a simpler and more independent structure. Multivariable models were developed using backward stepwise selection to identify independently significant predictors. Adjustment for baseline IOP was performed in the multivariable models. Outcomes of the analyses were reported in terms of odds ratios (OR), adjusted odds ratios (aOR), 95% confidence intervals (CI) and *p*‐values, highlighting significant findings at the 0.05 level. All statistical analyses were carried out using version 9.4 of the SAS software package (SAS Institute Inc.).

## 3 RESULTS

### 3.1 Demographic and clinical characteristics

**FIGURE 1:**
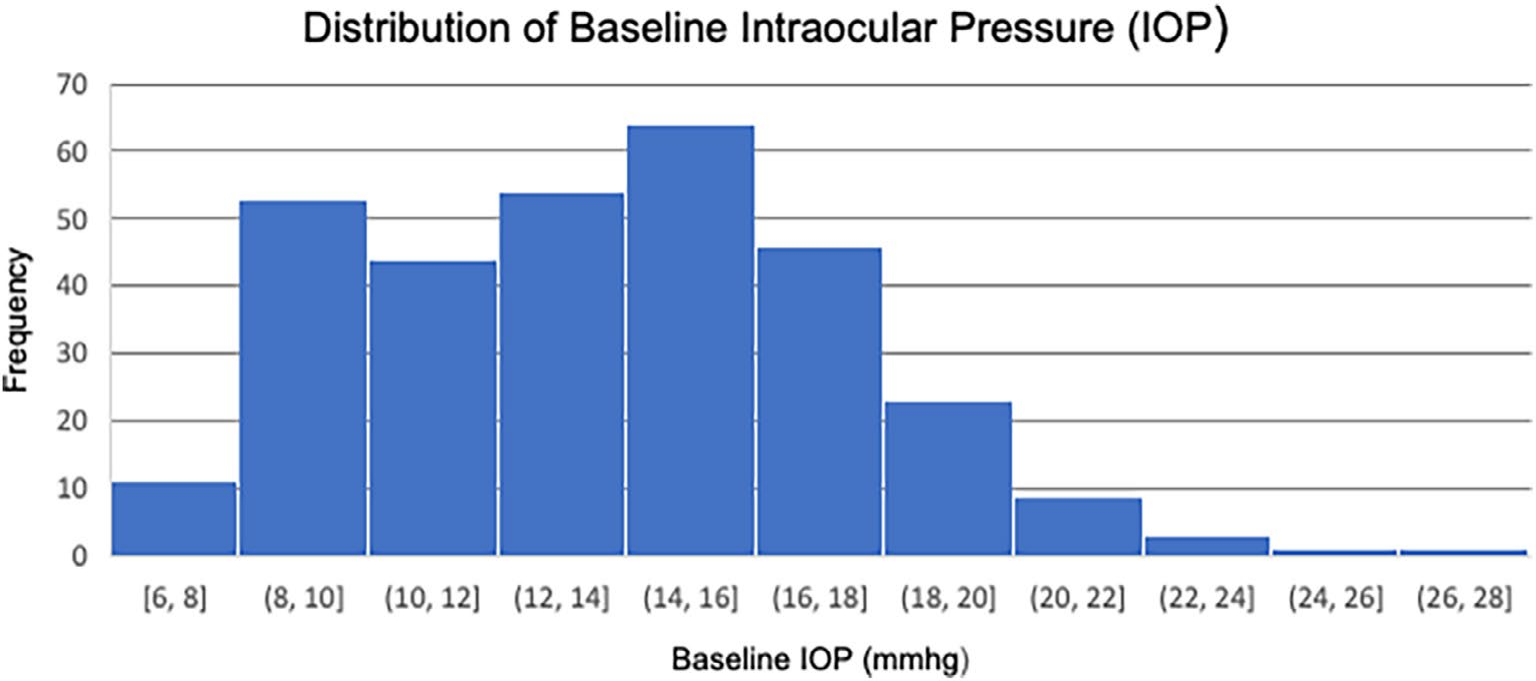
The histogram illustrates the frequency distribution of baseline intraocular pressure (IOP) values in the study population. The x‐axis represents baseline IOP values measured in mmHg, while the y‐axis indicates the frequency of observations within each IOP range.

The study encompassed 249 patients, 160 (64.3%) of whom were diagnosed with DME and 89 (35.7%) with RVO. The mean age was 71 years (39–96 years), with the majority (85.5%) being over 60 years old. Men were overrepresented (53.8%), particularly in the DME subgroup (58.1%). Baseline intraocular pressure (IOP) values are illustrated in Figure 1.

### 3.2 Intraocular pressure outcomes

**FIGURE 2:**
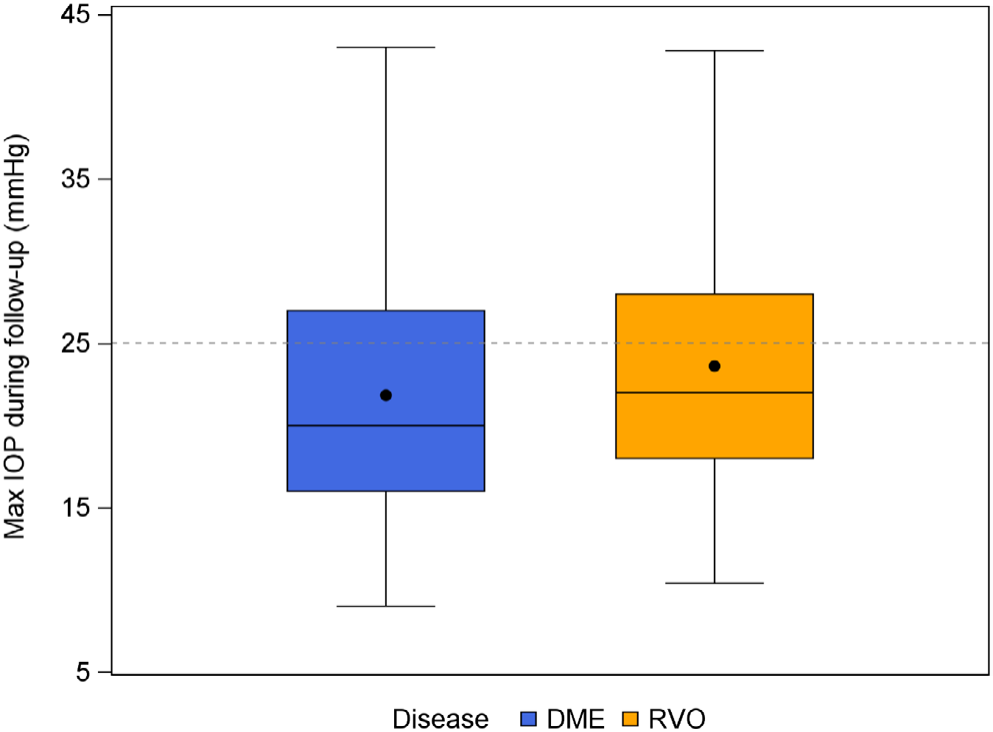
Box plot showing the intraocular pressure (IOP) response during the follow‐up period for patients with diabetic macular oedema (DME) and retinal vein occlusion (RVO) treated with Ozurdex® dexamethasone implants.

Of the total number of eyes, 117 (37.9%) developed SOHT, with 77 (35.5%) in the DME group and 40 (43.5%) in the RVO group. Throughout the follow‐up, the maximum IOP averaged 22.4 mmHg, with 68.0% maintaining an IOP under 25 mmHg. However, a substantial proportion (32.0%) of the eyes experienced a maximum IOP of 25 mmHg or more, indicating significant increases post‐treatment. The occurrence of IOP exceeding 30 mmHg was less common (18.1%), with 19.6% of eyes in the RVO group and 17.5% of eyes in the DME group experiencing such levels (Table 1). IOP responses in the DME and RVO groups are illustrated in Figure 2.

**TABLE 1:** Eye characteristics in the total group treated with dexamethasone implants, divided by diagnosis: diabetic macular oedema (DME) or macular oedema secondary to retinal vein occlusion (RVO).

Variable	Total (*n* = 309)	DME (*n* = 217)	RVO (*n* = 92)
Side			
Right	163 (52.8%)	119 (54.8%)	44 (47.8%)
Left	146 (47.2%)	98 (45.2%)	48 (52.2%)
IOP before treatment, mmHg	14.14 ± 3.73 14.00 (6.00–28.00)	14.17 ± 3.80 14.00 (6.00–28.00)	14.06 ± 3.59 13.00 (8.00–22.00)
IOP before treatment, dichotomized			
<15 mmHg	166 (53.7%)	111 (51.2%)	55 (59.8%)
≥15 mmHg	143 (46.3%)	106 (48.8%)	37 (40.2%)
Max IOP during follow‐up, mmHg	22.37 ± 7.62 21.00 (9.00–56.00)	21.84 ± 7.46 20.00 (9.00–49.00)	23.61 ± 7.87 22.00 (10.40–56.00)
Max IOP during follow‐up, dichotomized (cut‐off: 25 mmHg)			
<25 mmHg	210 (68.0%)	149 (68.7%)	61 (66.3%)
≥25 mmHg	99 (32.0%)	68 (31.3%)	31 (33.7%)
Max IOP during follow‐up, dichotomized (cut‐off: 30 mmHg)			
<30 mmHg	253 (81.9%)	179 (82.5%)	74 (80.4%)
≥30 mmHg	56 (18.1%)	38 (17.5%)	18 (19.6%)
Change in IOP from pre‐treatment to max during follow‐up, mmHg	8.23 ± 7.12 6.00 (−7.40 to 38.40)	7.67 ± 6.85 6.00 (−7.40 to 33.00)	9.56 ± 7.59 7.00 (−3.00 to 38.40)
Change in IOP from pre‐treatment to max during follow‐up, dichotomized			
<10 mmHg	208 (67.3%)	151 (69.6%)	57 (62.0%)
≥10 mmHg	101 (32.7%)	66 (30.4%)	35 (38.0%)

*Note*: Data are presented as mean ± standard deviation, median (minimum–maximum) or number (percentage).

Abbreviation: IOP, intraocular pressure.

### 3.3 Injection frequency

The average number of injections received was 3.2, with DME patients receiving an average of 3.28 injections and RVO patients receiving an average of 3.02 injections. The onset of SOHT generally occurred after an average of 2.6 injections. A detailed breakdown and further exploration of the impact of these injections can be found in the comprehensive data set outlined in Table S1.

### 3.4 Follow‐up time

**FIGURE 3:**
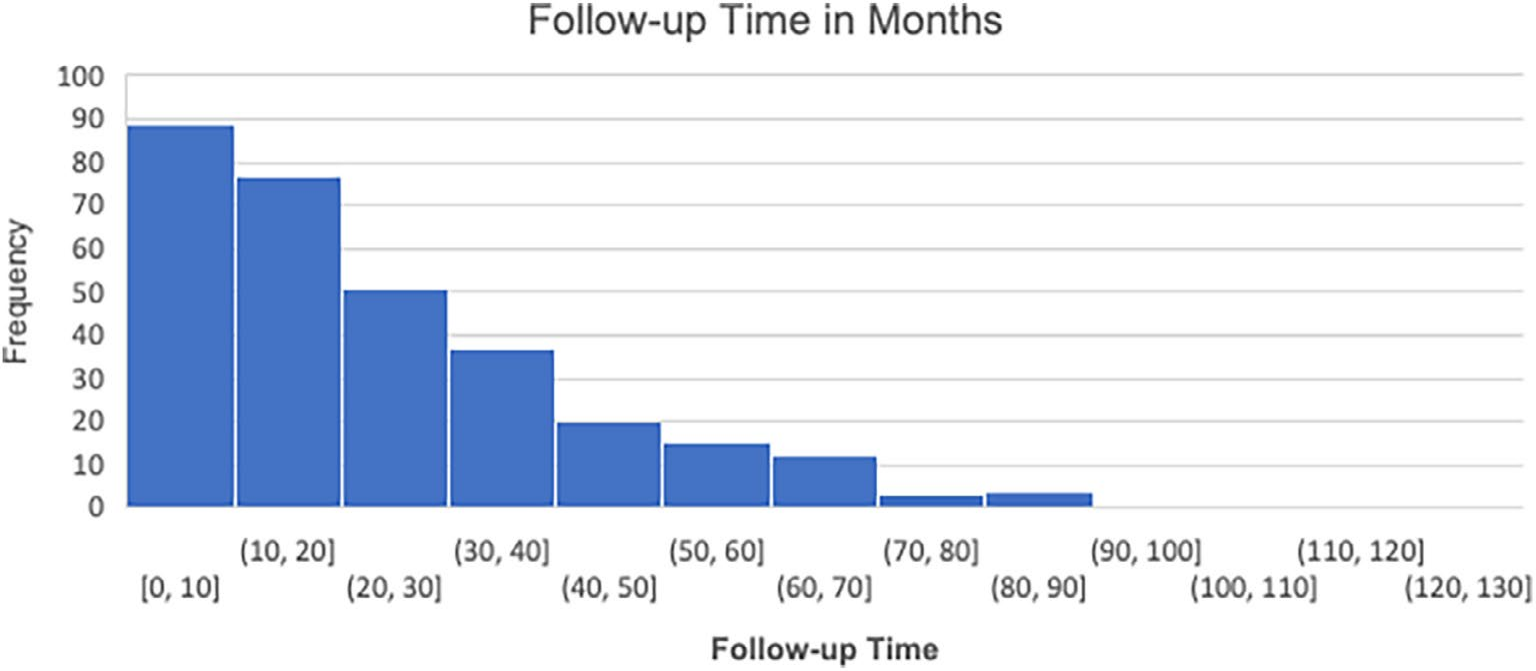
This histogram illustrates the distribution of follow‐up (in months) for the combined group of patients with diabetic macular oedema (DME) and macular oedema secondary to retinal vein occlusion (RVO). The x‐axis represents the follow‐up time in months, while the y‐axis shows the frequency of patients within each follow‐up time interval. The majority of patients have a follow‐up time between 0 and 20 months, with fewer patients followed for longer periods.

The average follow‐up time for all eyes was 23.5 months, with 25.4 months for the DME group and 19.0 months for the RVO group. A histogram of follow‐up time distribution is presented in Figure 3, illustrating the variability in follow‐up durations across the cohort.

### 3.5 Risk factors for SOHT

The development of SOHT was significantly and independently influenced by male gender and higher baseline IOP. Men were more than twice as likely as women to develop SOHT (aOR: 2.53, 95% CI: 1.51–4.25, *p* < 0.001), with 8% increased odds per unit increase in baseline IOP (aOR: 1.08, 95% CI: 1.01–1.15, *p* = 0.025). An increased number of Ozurdex® injections was also univariably associated with higher odds of developing SOHT (OR: 1.25, 95% CI: 1.12–1.39, *p* < 0.001). Age and disease (DME or RVO) were not statistically significantly associated with increased odds for SOHT (Table 2).

**TABLE 2:** Primary analysis of association between selected variables and ocular hypertension (SOHT).

Variable		Number of response eyes	Univariable analysis		Multivariable analysis	
			OR (95% Cl)	*p*‐Value	OR (95% Cl)	*p*‐Value
Age (per 1 year increase)	≤Median	60 (38.5%)				
	>Median	57 (37.3%)	0.98 (0.96–1.01)	0.19		
Age category	>60 years	98 (37.0%)	Reference			
	≤60 years	19 (43.2%)	1.44 (0.72–2.91)	0.30		
Sex	Female	39 (27.7%)	Reference			
	Male	78 (46.4%)	2.28 (1.36–3.81)	0.002	2.53 (1.51–4.25)	<0.001
Diagnosis	DME	77 (35.5%)	Reference			
	RVO	40 (43.5%)	1.36 (0.81–2.29)	0.25		
Baseline IOP	≤Median	57 (35.2%)				
	>Median	60 (40.8%)	1.05 (0.99–1.12)	0.10	1.08 (1.01–1.15)	0.025
Baseline IOP category (per 1 mmHg increase)	<15 mmHg	59 (35.5%)	Reference			
	≥15 mmHg	58 (40.6%)	1.03 (0.65–1.62)	0.91		
Total number of Ozurdex injections (per 1 injection increase)	≤Median	49 (30.8%)				
	>Median	68 (45.3%)	1.25 (1.12–1.39)	<0.0001		
Number of Ozurdex injections leading to SOHT or total (per 1 injection increase)	≤Median	72 (39.6%)				
	>Median	34 (29.3%)	1.04 (0.90–1.19)	0.61		

*Note*: Continuous variables were studied per 1 unit increase in the analyses. Numbers of events are descriptively presented in terms of below and above median for these variables.

Abbreviations: CI, confidence interval; DME, diabetic macular oedema; IOP, intraocular pressure; OR, odds ratio; RVO, retinal vein occlusion; SOHT, secondary ocular hypertension.

### 3.6 Treatment intervention for SOHT

**FIGURE 4:**
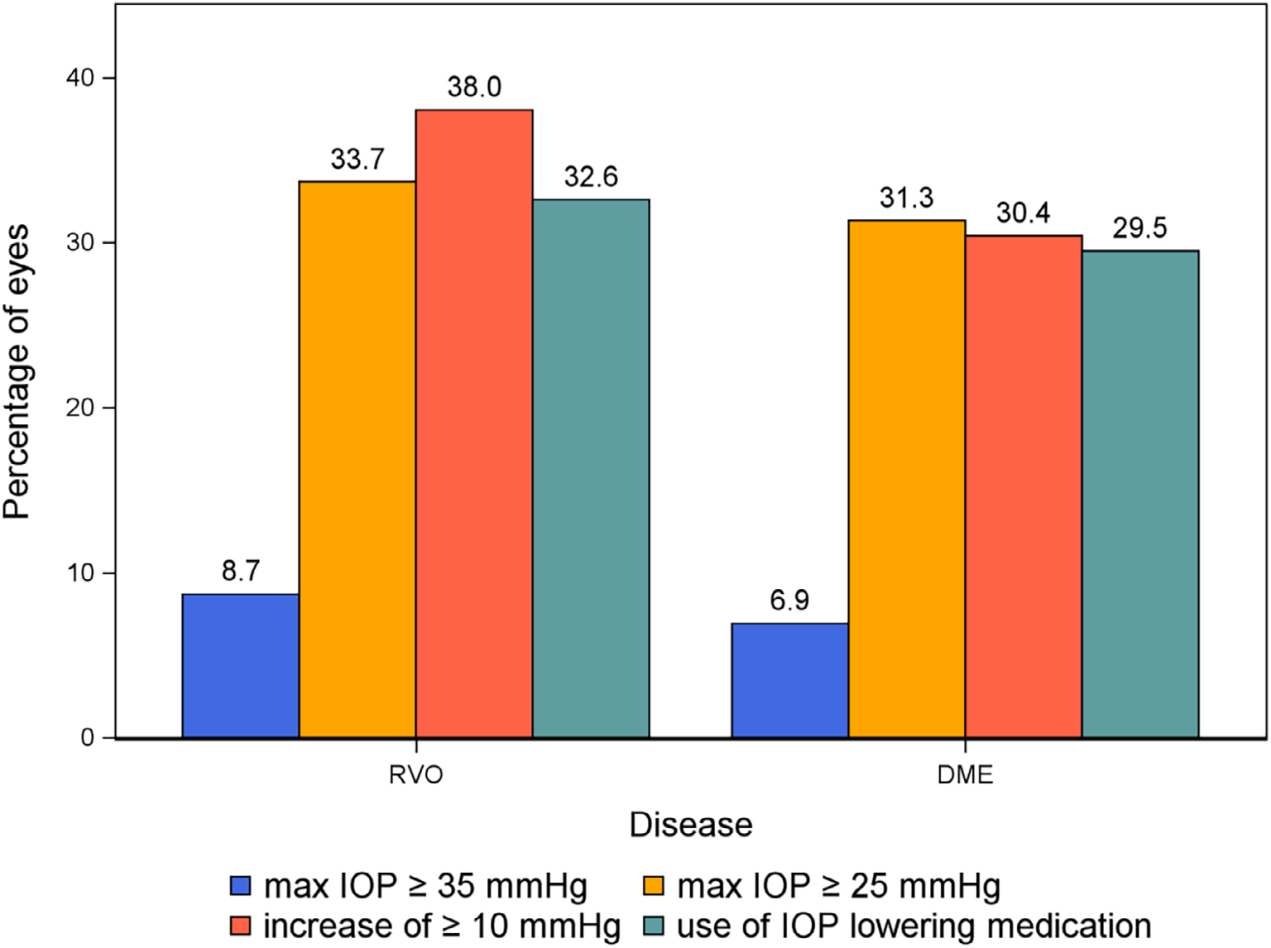
Percentage of eyes experiencing various intraocular pressure (IOP) outcomes, divided by pathology: Retinal vein occlusion (RVO) versus diabetic macular oedema (DME). Bars represent the proportion of eyes with maximum IOP ≥35 mmHg, maximum IOP ≥25 mmHg, an increase in IOP of ≥10 mmHg and use of IOP‐lowering medication, respectively. The data indicate higher rates of elevated IOP and use of IOP‐lowering treatments in eyes with RVO compared to those with DME.

Treatment initiation due to Ozurdex®‐induced SOHT was independently and significantly influenced by male gender and a higher number of Ozurdex® injections received, with men being nearly three times as likely as women to receive treatment (OR: 2.90, 95% CI: 1.62–5.19, *p* < 0.001) and having 7% higher odds of receiving intervention per increased number of injections provided (OR: 1.07, 95% CI: 1.01–1.14, *p* = 0.027) (Table 3). Among DME patients, male gender and the number of injections were significant predictors of treatment initiation. Male DME patients had higher odds of receiving treatment (OR: 3.64, 95% CI: 1.69–7.87, *p* = 0.001) and each additional Ozurdex® injection increased the odds by 10% (OR: 1.10, 95% CI: 1.02–1.18, *p* = 0.011) (Table S2.1). However, these associations were not statistically significant in RVO patients, where male gender and the number of injections did not significantly predict the initiation of treatment (Table S2.2). Of those considered to have elevated pressures, 30.5% were started on IOP‐lowering treatment (Figure 4). Prostaglandins were the most commonly used eye drop, administered in 64.9% of these cases, followed by beta blockers in 57.4% of cases (Table S1). Notably, invasive surgical interventions were not employed, and laser therapy was exceedingly rare, being applied to only one eye.

**TABLE 3:** Secondary analysis of association between selected variables and treatment provided for Ozurdex‐induced ocular hypertension (SOHT).

Variable		Number of response eyes	Univariable analysis		Multivariable analysis	
			OR (95% Cl)	*p*‐Value	OR (95% Cl)	*p*‐Value
Age	≤Median	46 (29.5%)				
	>Median	48 (31.6%)	0.99 (0.96–1.02)	0.43		
Age category	>60 years	80 (30.3%)	Reference			
	≤60 years	14 (31.8%)	1.19 (0.56–2.53)	0.65		
Sex	Female	29 (20.6%)	Reference			
	Male	65 (38.9%)	2.72 (1.53–4.83)	<0.001	2.90 (1.62–5.19)	<0.001
Disease	DME	65 (30.1%)	Reference			
	RVO	29 (31.5%)	1.03 (0.59–1.81)	0.91		
Baseline IOP	≤Median	34 (21.1%)				
	>Median	60 (40.8%)	1.12 (1.06–1.19)	<0.001		
Baseline IOP category	<15 mmHg	36 (21.8%)	Reference			
	≥15 mmHg	58 (40.6%)	1.70 (1.17–2.46)	0.005		
Total number of Ozurdex injections	≤Median	41 (25.9%)				
	>Median	53 (35.3%)	1.10 (1.03–1.17)	0.005	1.07 (1.01–1.14)	0.027
Number of Ozurdex injections leading to SOHT or total	≤Median	62 (34.3%)				
	>Median	32 (27.1%)	0.99 (0.93–1.04)	0.65		

*Note*: Continuous variables were studied per 1 unit increase in the analyses. Descriptively, number of events was presented below and above median for these variables.

Abbreviations: CI, confidence interval; DME, diabetic macular oedema; IOP, intraocular pressure; OR, odds ratio; RVO, retinal vein occlusion; SOHT, secondary ocular hypertension.

### 3.7 Treatment discontinuation due to elevated IOP

Baseline IOP ≥15 mmHg versus <15 mmHg was a significant predictor for the discontinuation of treatment due to elevated IOP across the entire cohort (OR: 1.85, 95% CI: 1.08–3.18, *p* = 0.025). This was particularly the case in DME patients (OR: 2.12, 95% CI: 1.12–4.03, *p* = 0.022). Conversely, in RVO patients, a higher number of injections leading to SOHT significantly decreased the odds of treatment discontinuation (OR: 0.50, 95% CI: 0.28–0.89, *p* = 0.018).

## 4 DISCUSSION

Our study presents a comprehensive analysis of SOHT incidence following the administration of Ozurdex® implants. It is novel in focusing on a Northern European cohort, an underrepresented population in steroid‐induced SOHT research. Additionally, it includes a relatively large number of eyes, enhancing the robustness and generalizability of the findings. Furthermore, the study spans a prolonged 8‐year observation period, providing valuable insights into the long‐term practice of Ozurdex® treatment in real‐world clinical settings. This is particularly important, as real‐world data often reveal patterns and challenges not evident in controlled trial settings. We observed that 37.9% of patients developed SOHT. Among all patients who received Ozurdex®, 30.5% received IOP‐lowering treatments. The remaining 7.4% of cases were managed with a watch‐and‐wait approach, based on the assessment of the treating physicians. In these cases, the elevated IOP levels decreased with observation alone, without the need for intervention. These findings indicate a higher incidence of SOHT compared to the existing literature and underscore significant risk factors such as male gender and higher baseline IOP (Badrinarayanan et al., 2022; Cho et al., 2024; Choi et al., 2019; Haller et al., 2011; Hemarat et al., 2018; Malclès et al., 2017; Sudhalkar et al., 2018). Additionally, 18.1% of patients experienced a maximum IOP of 30 mmHg or more. Such pressure levels are considered significantly high and typically necessitate immediate intervention rather than a wait‐and‐see approach.

We observed that men were more than twice as likely as women to develop SOHT, highlighting male gender as an important risk factor that has not been extensively emphasized in previous studies. Additionally, each unit increase in baseline IOP was associated with 8% increased odds of SOHT. These findings align with previous research, such as the SAFODEX study (Malclès et al., 2017), which also reported a higher SOHT incidence in men and a strong association with baseline IOP. A study by Cho et al. (2024) further supports our results by highlighting baseline IOP as a critical risk factor for steroid‐induced SOHT. Our study also examined the impact of baseline IOP on the discontinuation of Ozurdex® treatment due to elevated IOP. We found that a baseline IOP of 15 mmHg or higher significantly predicted the discontinuation of Ozurdex® treatment, particularly in patients with DME. This underscores the importance of baseline IOP as a predictive factor for treatment‐related adverse events. Given its significance, future studies should further explore the relationship between baseline IOP and treatment discontinuation to enhance patient management and treatment protocols.

Our data indicate that the risk of developing SOHT significantly increases with a higher number of Ozurdex® injections, generally occurring after an average of 2.6 injections. This emphasizes the necessity for vigilant monitoring of patients undergoing repeated treatments. Specifically, the cumulative risk analysis revealed that each additional Ozurdex® injection was associated with a 3% increase in the likelihood of developing SOHT, although this increase was not statistically significant. Similar findings have been reported in the literature, showing that multiple Ozurdex® injections can lead to mild to moderate increases in IOP, although severe spikes beyond 30 mmHg are less common (Bahadorani et al., 2018).

Patients with macular oedema secondary to RVO exhibited a 1.24 times higher risk of developing SOHT compared to those with DME, although this difference was not statistically significant (*p* = 0.42). This could be due to the underlying pathophysiological differences between these conditions, necessitating tailored monitoring and management strategies.

In our cohort, IOP was managed with topical treatments, with prostaglandin analogues used in 65% of cases. The most likely reason for prostaglandin analogues being the most commonly used eye drops is their favourable systemic safety profile, despite their theoretical risk of macular oedema. Notably, no patients required invasive surgical interventions, and laser therapy was rare, needed in only one of the eyes. These findings are consistent with other studies (Capone et al., 2014; Hemarat et al., 2018; Malclès et al., 2017), which also indicate that steroid‐induced SOHT is typically managed with topical ocular hypotensive agents.

A key methodological consideration is that during the study period, IOP was predominantly measured using rebound tonometry (iCare IC100, Icare Finland Oy) because this is the standard routine in our clinic, aligning with the demands of real‐world clinical settings and facilitating the management of high patient volumes. In cases where borderline IOP readings or additional confirmation were needed, clinicians occasionally chose to verify measurements using Goldmann applanation tonometry. Prior studies suggest that iCare tonometry may provide readings 1–3 mmHg higher than Goldmann applanation tonometry (Chen et al., 2019), and so these measurement differences should be accounted for when interpreting the results. However, it is important to note that in general, the same method was used consistently at both baseline and follow‐up, ensuring reliable comparative data.

The retrospective nature of this study introduces several limitations, including variability in follow‐up intervals, which may have resulted in missed transient IOP elevations. Additionally, the criteria for initiating IOP‐lowering treatment were not standardized and were left to the judgement of the treating ophthalmologists, potentially introducing variability in treatment initiation thresholds. Our initial plan to compare the effect of Ozurdex® on IOP in patients with pre‐existing glaucoma versus those without was hindered by the small number of glaucoma patients in our cohort (only two patients), reflecting a cautious approach in Swedish medical practice and meticulous patient selection to minimize the risk of complications. Furthermore, the study did not systematically exclude glaucoma using detailed optic disc assessments, visual field testing or OCT imaging, which limits our ability to fully differentiate steroid‐induced SOHT from undiagnosed glaucoma that may have developed due to secondary high IOP.

Clinicians should consider gender and baseline IOP when assessing the risk of SOHT in patients receiving Ozurdex® implants. The proactive management of elevated IOP with topical treatments, without the need for invasive interventions, underscores the importance of early detection and intervention. This approach helps alleviate exaggerated fears surrounding the use of intravitreal steroids. These findings are crucial for informing patients about the potential risks and benefits of treatment, helping them make informed decisions.

Further research is needed to explore the potential of prophylactic IOP‐lowering treatments in patients identified as being at high risk of SOHT. Additionally, prospective studies could provide more robust data on the long‐term management of IOP in patients receiving repeated Ozurdex® injections.

In conclusion, this study reinforces the importance of monitoring and managing IOP in patients receiving Ozurdex® implants, with particular attention to high‐risk groups identified by male gender and higher baseline IOP. Our findings contribute to the broader understanding of SOHT risks and management strategies, supporting the safe and effective use of intravitreal dexamethasone implants in clinical practice.

## FUNDING INFORMATION

This work was supported by a grant from the Gothenburg Society of Medicine. This organization had no role in the design or conduct of this research.

## CONFLICT OF INTEREST STATEMENT

The first author, Imadeddin Abu Ishkheidem, and the second author, Martin Breimer, have provided consultancy services to AbbVie on a topic unrelated to this study. Additionally, Imadeddin Abu Ishkheidem has provided consultancy services to Bayer, Roche, Théa Nordic, Topcon Scandinavia, Apellis, Novartis and OD‐OS. Martin Breimer has also provided consultancy services to Bayer, Novartis and Apellis. The other authors have no potential conflicts of interest to disclose.

## Supporting information


Table S1.
