# Supplementary material for: Secondary ocular hypertension as an adverse effect of treatment with intravitreal dexamethasone implant: A retrospective Swedish cohort study
Source: Acta Ophthalmol. 2025 Mar 5;103(6):699–706. doi: 10.1111/aos.17475 (PMC12340189; doi:10.1111/aos.17475)
Supplement: Supplementary file 1 — Table S1. [file AOS-103-699-s001.docx]

**Supplemental Material**

# **Supplementary Table 1.** Descriptive data of the primary and secondary variables in the total group receiving dexamethasone implant and divided by diagnosis: diabetic macular edema (DME) or macular edema secondary to retinal vein occlusion (RVO).

| **Variable** | **Total** n=309 | **DME** n=217 | **RVO** n=92 |
| --- | --- | --- | --- |
| **Primary variable** |  |  |  |
| Change in IOP from before treatment to max during follow-up | 8.23±7.12 6.00 (-7.40–38.40) | 7.67±6.85 6.00 (-7.40–33.00) | 9.56±7.59 7.00 (-3.00–38.40) |
| **Secondary variables** |  |  |  |
| Ocular hypertension (SOHT) |  |  |  |
| No | 192 (62.1%) | 140 (64.5%) | 52 (56.5%) |
| Yes | 117 (37.9%) | 77 (35.5%) | 40 (43.5%) |
| Need for pressure-lowering treatment |  |  |  |
| No | 214 (69.5%) | 151 (69.9%) | 63 (68.5%) |
| Yes | 94 (30.5%) | 65 (30.1%) | 29 (31.5%) |
| Use of pressure-lowering laser treatment |  |  |  |
| No | 308 (99.7%) | 217 (100.0%) | 91(98.9%) |
| Yes | 1 (0.3%) | 0 (0.0%) | 1 (1.1%) |
| Use of pressure-lowering medication |  |  |  |
| No | 215 (69.6%) | 153 (70.5%) | 62 (67.4%) |
| Yes | 94 (30.4%) | 64 (29.5%) | 30 (32.6%) |
| Prostaglandin |  |  |  |
| No | 33 (35.1%) | 23 (35.9%) | 10 (33.3%) |
| Yes | 61 (64.9%) | 41 (64.1%) | 20 (66.7%) |
| Beta blockers |  |  |  |
| No | 40 (42.6%) | 26 (40.6%) | 14 (46.7%) |
| Yes | 54 (57.4%) | 38 (59.4%) | 16 (53.3%) |
| Carbonic anhydrase inhibitors |  |  |  |
| No | 60 (63.8%) | 42 (65.6%) | 18 (60.0%) |
| Yes | 34 (36.2%) | 22 (34.4%) | 12 (40.0%) |
| Alpha agonist |  |  |  |
| No | 83 (90.2%) | 55 (88.7%) | 28 (93.3%) |
| Yes | 9 (9.8%) | 7 (11.3%) | 2 (6.7%) |
| Treatment discontinuation |  |  |  |
| No | 242 (78.3%) | 166 (76.5%) | 76 (82.6%) |
| Yes | 67 (21.7%) | 51 (23.5%) | 16 (17.4%) |
| Data are presented as mean±standard deviation, median (minimum–maximum), or number (percentage).  Abbreviations: IOP, intraocular pressure. | | | |

# **Supplementary Table 2**.**1.** Secondary analysis of associations between selected variables and treatment provided for Ozurdex-induced ocular hypertension (SOHT) in patients with diabetic macular edema.

|  | | | **Univariable analysis** | | **Multivariable analysis** | |
| --- | --- | --- | --- | --- | --- | --- |
| **Variable** |  | **Number of response eyes (%)** | **OR (95% Cl)** | **p-value** | **OR (95% Cl)** | **p-value** |
| Age | ≤median | 31 (26.7) |  |  |  |  |
|  | >median | 34 (34.0) | 0.98 (0.95–1.02) | 0.35 |  |  |
| Age category | >60 years | 53 (29.6%) | Reference |  |  |  |
|  | ≤60 years | 12 (32.4%) | 1.29 (0.55–3.03) | 0.55 |  |  |
| Sex | Female | 16 (17.6%) | Reference |  |  | . |
|  | Male | 49 (39.2%) | 3.27 (1.53–7.01) | 0.002 | 3.64 (1.69–7.87) | 0.001 |
| Baseline IOP | ≤median | 21 (19.4%) |  |  |  |  |
|  | >median | 44 (40.7%) | 1.13 (1.05–1.20) | <0.001 |  |  |
| Baseline IOP category | <15 mmHg | 22 (20.0%) | Reference |  |  |  |
|  | ≥15 mmHg | 43 (40.6%) | 1.86 (1.18–2.94) | 0.008 |  |  |
| Total number of Ozurdex injections | ≤median | 25 (22.7%) |  |  |  |  |
|  | >median | 40 (37.7%) | 1.11 (1.03–1.20) | 0.005 | 1.10 (1.02–1.18) | 0.011 |
| Number of Ozurdex injections leading to SOHT or total | ≤median | 39 (31.2%) |  |  |  |  |
|  | >median | 26 (29.9%) | 1.00 (0.94–1.07) | 0.92 |  |  |
| Continuous variables were studied per 1 unit increase in the analyses. Descriptively, number of events was presented below and above median for these variables.  Abbreviations: CI, confidence interval; IOP: intraocular pressure; OR, odds ratio;SOHT, ocular hypertension. | | | | | | |

# **Supplementary Table 2.2.** Secondary analysis of association between selected variables and treatment provided for Ozurdex-induced ocular hypertension (SOHT) in patients with macular edema secondary to retinal vein occlusion.

|  | | | **Univariable analysis** | | **Multivariable analysis** | |
| --- | --- | --- | --- | --- | --- | --- |
| **Variable** |  | **Number of response eyes**  **(%)** | **OR (95% Cl)** | **p-value** | **OR (95% Cl)** | **p-value** |
| Age | ≤median | 17 (35.4%) |  |  |  |  |
|  | >median | 12 (27.3%) | 1.00 (0.95–1.05) | 0.89 |  |  |
| Age category | >60 years | 27 (31.8%) | Reference |  |  |  |
|  | ≤60 years | 2 (28.6%) | 0.91 (0.17–5.02) | 0.92 |  |  |
| Sex | Female | 13 (26.0%) | Reference |  |  | . |
|  | Male | 16 (38.1%) | 2.15 (0.86–5.40) | 0.10 | 2.29 (0.80–6.56) | 0.12 |
| Baseline IOP | ≤median | 12 (25.5%) |  |  |  |  |
|  | >median | 17 (37.8%) | 1.00 (1.00–1.00) | 0.47 |  |  |
| Baseline IOP category | <15 mmHg | 14 (25.5%) | Reference |  |  |  |
|  | ≥15 mmHg | 15 (40.5%) | 1.00 (1.00–1.00) | 0.52 |  |  |
| Total number of Ozurdex injections | ≤median | 16 (33.3%) |  |  |  |  |
|  | >median | 13 (29.5%) | 1.07 (0.89–1.28) | 0.47 | 1.34 (0.52–3.46) | 0.54 |
| Number of Ozurdex injections leading to SOHT or total | ≤median | 23 (41.1%) |  |  |  |  |
|  | >median | 6 (19.4%) | 1.00 (1.00–1.00) | 0.61 |  |  |
| Continuous variables were studied per 1 unit increase in the analyses. Descriptively, number of events was presented below and above median for these variables.  Abbreviations: CI, confidence interval; IOP: intraocular pressure; OR, odds ratio; SOHT, ocular hypertension. | | | | | | |
